# Frequency and Profiles of Drug Combinations Constituting the Triple Whammy in Japan: An Analysis of a Patient Estimation Database

**DOI:** 10.3390/pharmacy14050117

**Published:** 2026-08-08

**Authors:** Mari Maese, Nozomi Ito, Haruka Shiokawa, Shingo Kondo, Yuko Okamoto, Hiroki Iwata, Shunsuke Urushibata, Katsunori Yamaura

**Affiliations:** 1 Division of Social Pharmacy, Center for Social Pharmacy and Pharmaceutical Sciences, Faculty of Pharmacy, Keio University, Tokyo 105-8512, Japan; mari.m-1128@keio.jp (M.M.); nozomiito1011@keio.jp (N.I.); haruka.shiokawa@keio.jp (H.S.); okamoto-yk@keio.jp (Y.O.); iwahiro@keio.jp (H.I.); yamaura-kt@keio.jp (K.Y.); 2 Keio University Community Pharmacy, Tokyo 105-8512, Japan; 3 Japan Pharmacy Collaboration for Heart Failure, Kanagawa 250-0011, Japan; shunsuke.urushibata@jpchf.or.jp

**Keywords:** renin–angiotensin system inhibitor, non-steroidal anti-inflammatory drug, diuretic, acute kidney injury

## Abstract

“Triple whammy” prescriptions, combining non-steroidal anti-inflammatory drugs (NSAIDs), renin–angiotensin system (RAS) inhibitors, and diuretics, increase acute kidney injury (AKI) risk. To clarify the frequency and profiles of these prescriptions and identify vulnerable populations, we analyzed the AHI partners database to estimate the number of individuals prescribed these three drug classes. In the single-agent analysis, loxoprofen was the most commonly prescribed NSAID (66.3%), olmesartan (19.4%) and telmisartan (15.9%) were the predominant RAS inhibitors, and furosemide (19.6%) and spironolactone (16.0%) were the most frequently used diuretics. Dual-drug combinations showed patterns consistent with the single-agent results for NSAIDs and diuretics. By contrast, sacubitril/valsartan was the most common RAS inhibitor when combined with diuretics, frequently utilized for heart failure management. In triple whammy prescriptions, NSAIDs and diuretics patterns mirrored those of single-agents. The annual number of triple whammy prescriptions showed a statistically significant downward trend over the study period by the Mann–Kendall trend test (*p* = 0.048). Notably, sacubitril/valsartan was the leading RAS inhibitor (46/199 patients, 23%), showing a higher proportion than its single-agent use (7.4%). Heart failure patients prescribed these two causative drugs are highly vulnerable to “triple whammy” prescriptions. Awareness of inadvertent NSAID additions is warranted to mitigate potential AKI risks.

## 1. Introduction

Acute kidney injury (AKI) is a multifactorial disease caused by a rapid decline in kidney function over a short period [1]. In addition to increasing mortality rates and medical costs, AKI can lead to the development of heart failure, stroke, dementia, and frailty. According to the widely accepted criteria published by the Kidney Disease–Improving Global Outcomes (KDIGO) organization, an AKI diagnosis is based on any of the following: an increase in serum creatinine (Scr) of 0.3 mg/dL or higher within 48 h; an increase in Scr to 1.5 times the baseline or more within 7 days; or a urine volume of less than 0.5 mL·kg^−1^·h^−1^ for 6 h [2]. Notably, pharmacotherapy is recognized as one of the factors contributing to the development of AKI [1]. Although the term “triple whammy”—referring to the combined use of non-steroidal anti-inflammatory drugs (NSAIDs), renin–angiotensin system (RAS) inhibitors, and diuretics—was first established globally in 2000 by Thomas [3], it has become widely recognized in numerous clinical reports over the past decade as a significant contributor to AKI development. A recent global meta-analysis involving over 2 million participants reported that patients exposed to the triple whammy have an approximately 2.01-fold higher risk of developing AKI compared to those not exposed [4]. Actually, a follow-up study of outpatients prescribed diuretics, RAS inhibitors, or NSAIDs found that while the overall AKI incidence was 3.40 cases per 1000 users per year, the rate was significantly higher among those using a triple combination of RAS inhibitors, diuretics, and NSAIDs at 8.82 cases per 1000 users [5]. The increased risk of AKI is attributed to the synergistic reduction in renal blood flow and glomerular filtration pressure caused by the combination of these three classes of drugs [6,7]. Mechanistically, NSAIDs reduce renal blood flow by inhibiting cyclooxygenase and decreasing prostaglandin production, which leads to vasoconstriction. A meta-analysis of community-dwelling individuals revealed that NSAID use was associated with a 1.73-fold increased risk of AKI compared with non-use of the drug [8]. RAS inhibitors expand the efferent arterioles by blocking angiotensin II, thereby decreasing the glomerular filtration rate. Additionally, diuretics reduce renal blood flow by promoting fluid excretion. Our previous database analysis of patients on diuretics revealed that hypokalemia was more likely to occur in female patients with reduced renal function [9]. In 2018, the Study Group on the Appropriate Medication for Elderly Patients, established by the Ministry of Health, Labour and Welfare (MHLW) of Japan, recommended that triple whammy prescriptions be avoided owing to the increased risk of renal function decline and hyponatremia. However, these drugs play essential therapeutic roles in clinical practice.

Among the RAS inhibitors, sacubitril/valsartan sodium hydrate has emerged as an important therapeutic option for heart failure, with evidence supporting its improvement of prognosis [10,11,12]. This agent is a combination of sacubitril (a prodrug with neprilysin-inhibiting activity) and valsartan (an angiotensin II receptor blocker). Accordingly, it exhibits pharmacological properties distinct from those of conventional RAS inhibitors. In Japan, the number of patients with heart failure is increasing annually and estimated to reach approximately 1.3 million by 2030 [13]. This situation, referred to as the “heart failure pandemic,” has become a major public health challenge. Current heart failure guidelines recommend the “fantastic four” (ACE inhibitors/ARBs/angiotensin receptor-neprilysin inhibitors, beta-blockers, mineralocorticoid receptor antagonists, and sodium-glucose cotransporter 2 inhibitors) for the treatment of advanced heart failure and specific phenotypes. Additionally, although diuretics lack definitive evidence for improving survival, their use under hemodynamic monitoring guidance has been suggested to help prevent the exacerbation of heart failure [14,15,16]. Because diuretics and RAS inhibitors are commonly used in patients with advanced heart failure, the addition of NSAIDs may inadvertently result in the triple whammy. Although the avoidance of this remains crucial, attention must also be paid to preventing reactions to each drug.

In a 2017 analysis of the medication data of 246,721 individuals registered in the Japan Medical Data Center claims database, triple whammy prescriptions were identified in 730 (0.3%) patients [17]. However, because those data were from 2017, before the approval and widespread use of sacubitril/valsartan sodium hydrate in Japan, current triple whammy prescribing patterns in routine clinical practice may have changed since then. Furthermore, the September 2021 approval of sacubitril/valsartan sodium hydrate for hypertension treatment implies a projected increase in its clinical use. To mitigate the risk associated with triple whammy prescriptions, proactive intervention by pharmacists—including prescription proposals or implementing rigorous monitoring—is essential. However, the prescribing patterns of drug classes and their combinations that constitute the triple whammy in real-world clinical practice have not been fully elucidated.

Therefore, this study was conducted to analyze the real-world prescribing patterns and constituent drug combinations of the triple whammy in Japan using a patient estimation database. The findings of this study contribute to pharmacovigilance and the optimization of prescribing practices by increasing awareness of the triple whammy effect and providing information to support its prevention and pharmacist-led monitoring.

## 2. Materials and Methods

### 2.1. Data Source

Data were obtained from the AHI partners database, an online search system (AHI partners Inc., Tokyo, Japan) [18]. The database is a nationwide patient estimation database constructed based on the Survey of Medical Care Benefit Status published by the MHLW of Japan. This survey covers approximately 99% of the Japanese population, across multiple public health insurance systems. To estimate the nationwide patient numbers, the database adjusted the raw claims data using expansion coefficients derived from the MHLW reports. These coefficients were calculated by considering factors such as prefecture, age group, medical fee categories, and disease-specific age distributions. Through this calibration process, the database provides data comparable in scope and representativeness to that of the National Database of Japan. The database provides information on patient demographics, clinical diagnoses coded according to the ICD-10, clinical departments, and prescribed drugs in routine clinical practice.

### 2.2. Drug Definitions

The target drugs in this study were defined using Anatomical Therapeutic Chemical (ATC) classification codes and classified as NSAIDs (M01A), RAS inhibitors (C09A, C09C, C09D, and C09X), or diuretics (C01D, C03A, C03B, C03D, and C03X) (Table 1). Additionally, combination drugs were defined and categorized as RAS inhibitors + diuretics (C09D) or RAS inhibitors + calcium channel blockers + diuretics (C09D) (Table 2). The ATC codes were described at the third level (pharmacological subgroup) to enable consistent classification across the drug classes. With regard to the search criteria, only oral drugs were included, whereas injections and topical drugs were excluded. Generic names were used instead of brand names for calculation of the prescription counts.

The drugs were classified on the basis of Anatomical Therapeutic Chemical-10 (ATC-10) codes: non-steroidal anti-inflammatory drugs (NSAIDs) (M01A), renin–angiotensin system (RAS) inhibitors (C09A, C09C, C09D, and C09X), and diuretics (C01D, C03A, C03B, C03C, C03D, and C03X).

The drugs were classified on the basis of ATC-10 codes: RAS inhibitors + diuretics (C09D) and RAS inhibitors + Ca^2+^ blocker + diuretics (C09D).

### 2.3. Data Analysis

Trends in the estimated total number of patients treated with each drug were analyzed from 2016 to 2024. Each year was defined as the period from July to June of the following year; therefore, the latest data covered the period up to June 2025. To avoid bias caused by fluctuations in prescription counts within a single year, the number of patients prescribed each categorized drug was calculated over a 3-year period. Specifically, “drug use” was defined as receiving at least one prescription for diuretics, RAS inhibitors, or NSAIDs within the analysis period. In contrast, “drug combination use” was defined as the concurrent prescription of specific two- or three-drug combinations, identified by an overlap in the prescription duration of the respective medications at least once during the 3-year observation window. By requiring simultaneous prescriptions rather than simple overlapping at different points within the 3-year period, this study aimed to identify the population effectively exposed to the pharmacological risks of combination therapy. Owing to the small number of patients who were prescribed a triple whammy combination and the potential for large estimation errors, annual trends were calculated using the actual number of patients registered in the database. To indicate the precision of the national estimates, trends in the number of prescribed patients for each therapeutic category were reported using 9-year annual averages and their 95% confidence intervals (CIs), calculated based on Student’s t-distribution (df = 8). The temporal trajectory of triple whammy prescriptions was evaluated using the non-parametric Mann–Kendall trend test. Furthermore, two-proportion z-tests were performed to compare the proportion of specific drugs between single-agent use and drug combination use. All statistical calculations and data visualizations were performed using R Studio software version 2026.04.0+526 (Posit Software, Boston, MA, USA).

### 2.4. Ethical Considerations

The database used in this study is fully anonymized, thus precluding the identification of individual medication records and rendering the collection of detailed patient background information unfeasible. Consequently, while this study was conducted in accordance with the principles of the Declaration of Helsinki, an ethical review regarding the management of personal information was not applicable to this study in accordance with the Ethical Guidelines for Medical and Biological Research Involving Human Subjects in Japan.

## 3. Results

Extending beyond the scope of previous studies, the comprehensive analysis in this study incorporated all available single-agent drugs within the NSAID, RAS inhibitor, and diuretic classes to identify the estimated total number of patients with triple whammy prescriptions on the basis of the most recent multi-year data. Table 1 lists the target single-agent drugs classified by ATC codes, where 30 NSAIDs, 21 RAS inhibitors, and 17 diuretics were identified as being associated with triple whammy prescriptions. Furthermore, this analysis included five drug combinations consisting of RAS inhibitors and diuretics. A triple-combination drug containing an RAS inhibitor, a diuretic, and a calcium channel blocker was also incorporated (Table 2).

### 3.1. Annual Trends in the Estimated Number of Patients Prescribed Each Therapeutic Category

**Figure 1:**
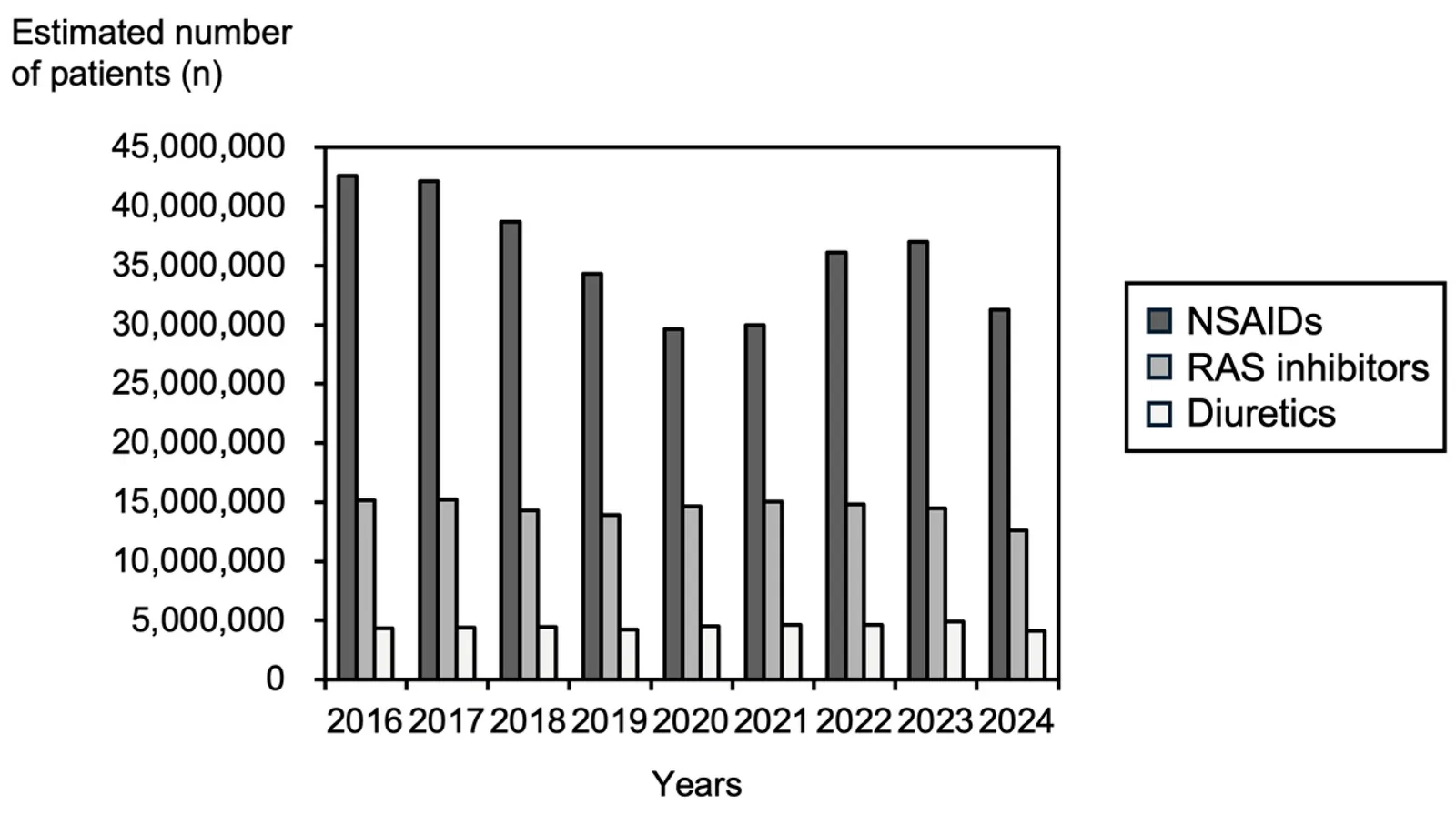
Trends in the number of patients prescribed each drug class. To avoid discrepancies due to the double counting of classifications in combination drugs, the data represent the estimated number of patients prescribed single-agent drugs, NSAIDs, RAS inhibitors, and diuretics (sum of the drugs listed in Table 1). Each year covers the period from July to June of the following year.

From July 2016 to June 2025, the average annual numbers with 95% CIs of patients prescribed NSAIDs, RAS inhibitors, and diuretics were 35,756,817 (95% CI: 32,000,103–39,513,532), 14,481,681 (95% CI: 13,861,973–15,101,388), and 4,468,199 (95% CI: 4,290,492–4,645,906), respectively (Figure 1). In the latest year (2024), the patient counts were 31,276,066 for NSAIDs, 12,646,774 for RAS inhibitors, and 4,109,788 for diuretics, with NSAIDs being the most frequently prescribed drugs in all years. The number of patients in each year was similar to that in the other years across the three drug classes, indicating a plateau. However, the number of patients prescribed NSAIDs decreased by 13.6% in 2020 compared with the previous year, remained nearly constant in 2021, and increased by 20.5% in 2022. This decline over the years is consistent with the timing of the coronavirus disease 2019 (COVID-19) pandemic.

### 3.2. Estimated Number of Patients Prescribed the Target Single-Agent Drugs

**Figure 2:**
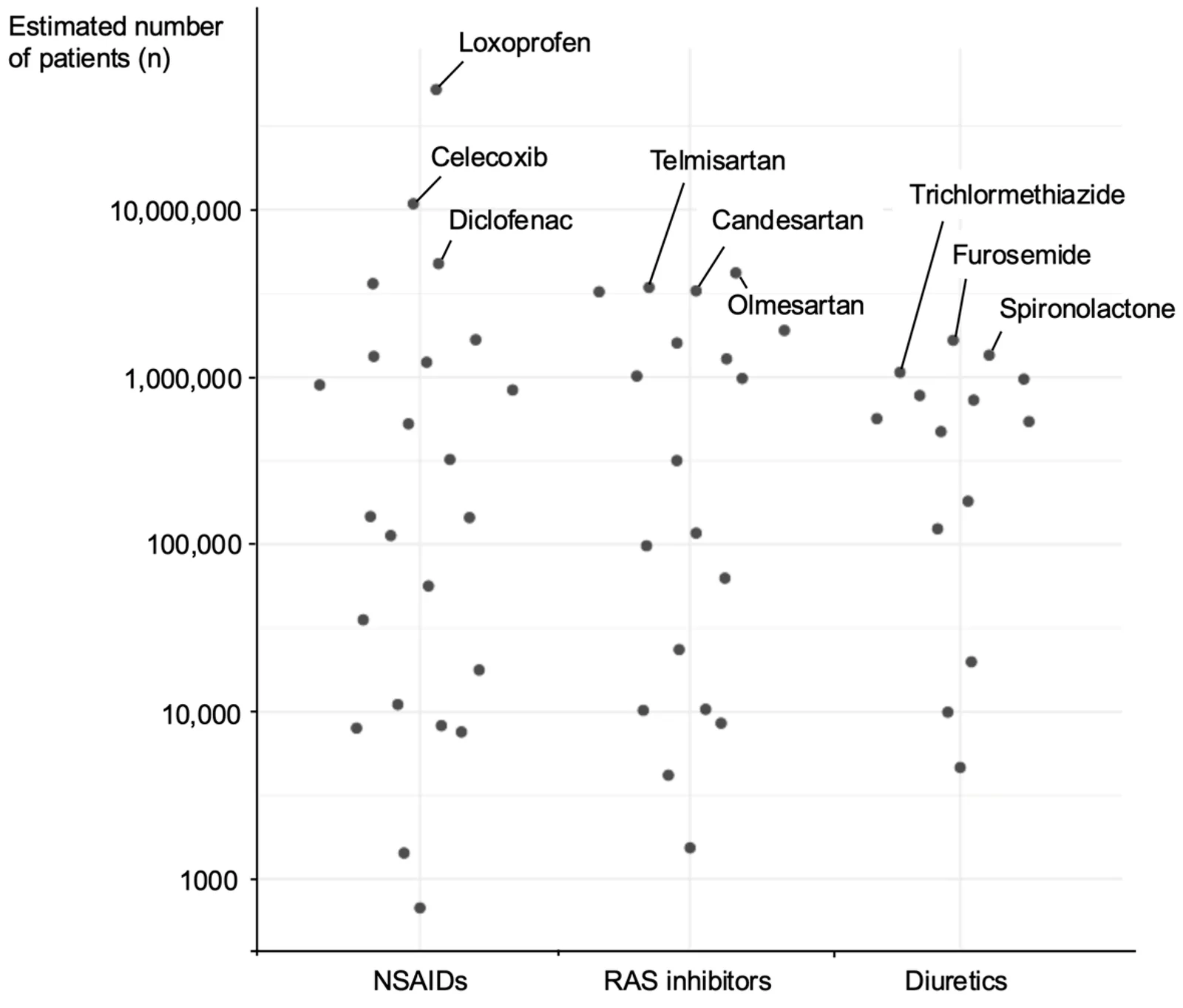
Number of patients prescribed single-agent drugs from the three classes. Estimated number of patients over the past 3 years, from July 2022 to June 2025. Injections or topical drugs were excluded. For each drug class, the top three most frequently prescribed drugs are displayed.

Over the 3-year period from July 2022 to June 2025, the most frequently prescribed NSAIDs were loxoprofen sodium hydrate (52,040,950 patients), celecoxib (10,815,500 patients), and diclofenac sodium (4,747,014 patients) (Figure 2). The individuals prescribed these three drugs represented 66.3%, 13.8%, and 6.0% of all NSAID-prescribed patients, respectively, with the top three drugs collectively accounting for 86.1% of the total NSAIDs prescribed. The most common RAS inhibitors were olmesartan medoxomil (4,180,448 patients; 19.4%), telmisartan (3,423,481 patients; 15.9%), and candesartan cilexetil (3,265,277 patients; 15.2%). The leading diuretic drugs were furosemide (1,653,440 patients; 19.6%), spironolactone (1,347,960 patients; 16.0%), and trichlormethiazide (1,064,811 patients; 12.6%).

### 3.3. Estimated Number of Patients Prescribed Two-Drug Class Combinations

**Figure 3:**
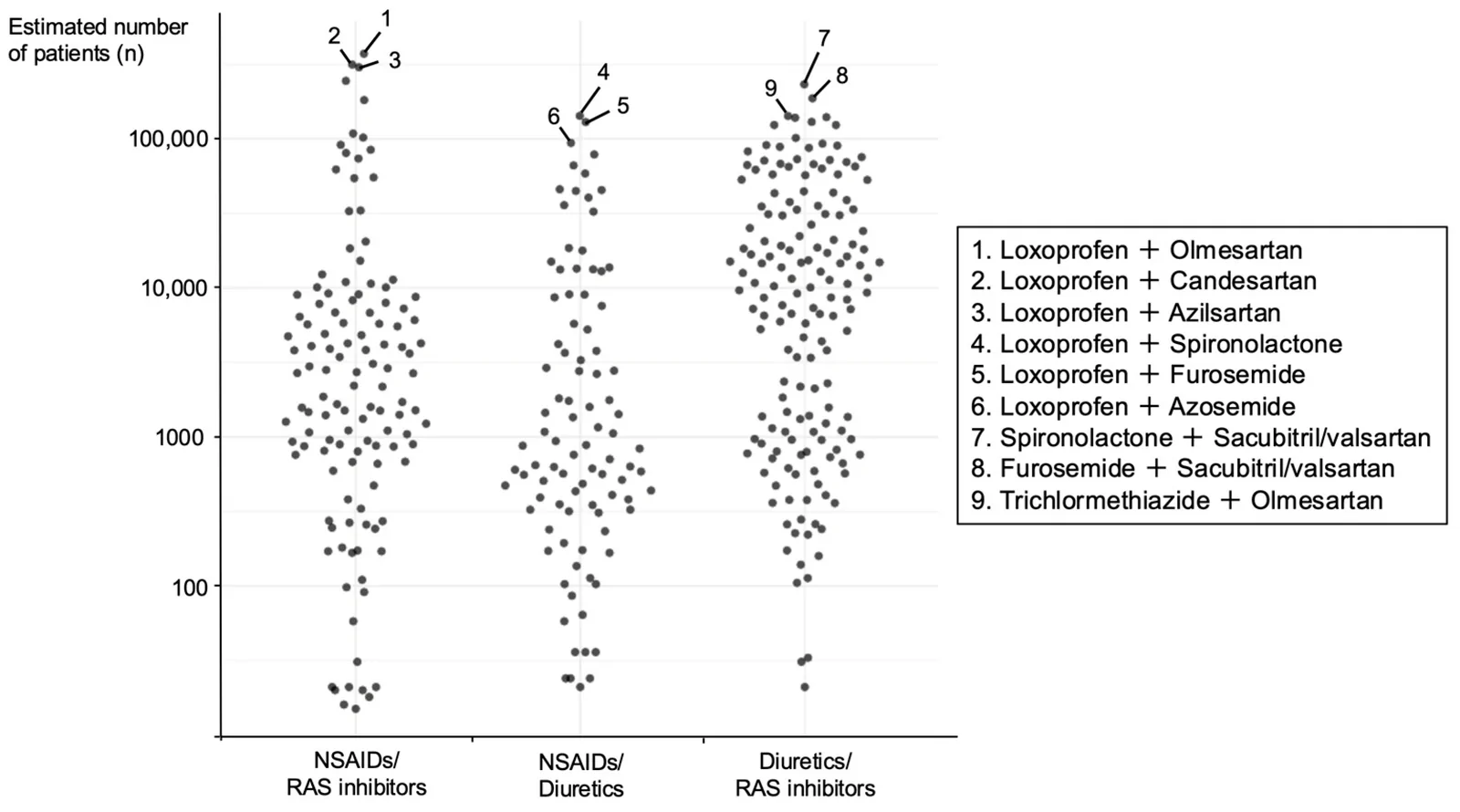
Number of patients prescribed dual-combinations of the three drug classes. Estimated number of patients prescribed combinations of two drug classes among NSAIDs, RAS inhibitors, and diuretics. The data cover the period from July 2022 to June 2025. Injections or topical drugs were excluded. For each combination of drug classes, the top three most frequently prescribed drug pairs are displayed.

The estimated numbers of patients prescribed combinations of NSAIDs and RAS inhibitors, NSAIDs and diuretics, and diuretics and RAS inhibitors over the last 3 years are shown in Figure 3. Among the 123 identified combinations of NSAIDs and RAS inhibitors, those containing loxoprofen sodium hydrate accounted for 70.0% of all patients, despite representing only 16 specific drug pairs. The three most frequent two-drug prescriptions were loxoprofen in combination with olmesartan medoxomil (372,343 patients), candesartan cilexetil (314,918 patients), or azilsartan (301,789 patients). Similarly, the highest number of prescriptions with an NSAID and a diuretic combined was observed for loxoprofen sodium hydrate in combination with spironolactone (142,613 patients), followed by its combination with furosemide (129,566 patients) or azosemide (93,848 patients). Of the 94 combinations identified, 13 containing loxoprofen sodium hydrate accounted for 64.9% of the total. Notably, this specific NSAID was predominant in these combinations, mirroring the trend observed for RAS inhibitors. By contrast, the most frequent combination of diuretics and RAS inhibitors was spironolactone with sacubitril/valsartan sodium hydrate (232,102 patients), followed by furosemide with sacubitril/valsartan sodium hydrate (187,005 patients) and trichlormethiazide with olmesartan medoxomil (142,199 patients). Prescriptions containing sacubitril/valsartan sodium hydrate, a therapeutic agent for heart failure, accounted for 25.2% of all prescriptions.

### 3.4. Estimated Number of Patients Prescribed Combination Drugs Representing Two Drug Classes

Combination drugs containing both a RAS inhibitor and a diuretic correspond to the concurrent use of two drug classes within a single-agent. Among the six combination drugs identified, losartan potassium/hydrochlorothiazide accounted for the highest number of patients to which it was prescribed (385,789 patients). This was followed by telmisartan/hydrochlorothiazide (296,276 patients), candesartan cilexetil/hydrochlorothiazide (225,158 patients), valsartan/hydrochlorothiazide (132,159 patients), irbesartan/trichlormethiazide (34,442 patients), and telmisartan/amlodipine besilate/hydrochlorothiazide (23,621 patients). Importantly, for patients taking any of these combination drugs, the addition of a single NSAID would constitute a triple whammy prescription.

### 3.5. Annual Trends in Triple Whammy Prescriptions and Prescribing Patterns by Drug Class

**Figure 4:**
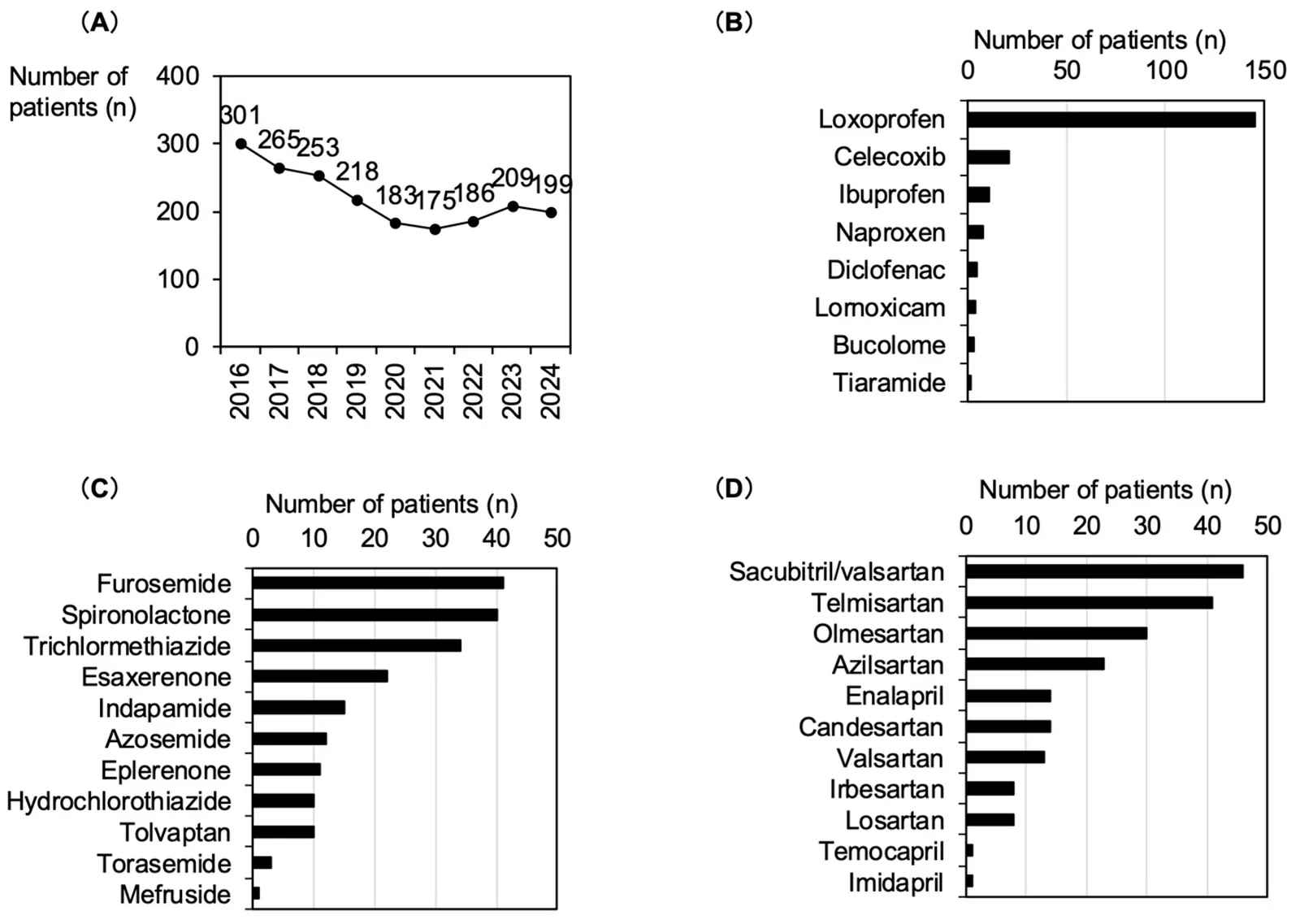
Trends and constituent drug profiles of triple whammy prescriptions. To avoid estimation errors due to the small sample size, the data represent the actual number of patients registered in the database. (**A**) Trends in patients receiving triple whammy prescriptions between 2016 and 2024. (**B**–**D**) Detailed prescribing patterns among patients with triple whammy prescriptions in the most recent year: (**B**) NSAIDs, (**C**) diuretics, and (**D**) RAS inhibitors.

Analysis of the trends in the number of patients receiving triple whammy prescriptions revealed that the annual average number over the 9-year period was 221 (Figure 4A). The annual number of triple whammy prescriptions showed a statistically significant downward trend over the study period according to the Mann–Kendall trend test (*p* = 0.048). Analysis of the detailed prescription status using actual data from the most recent year revealed that loxoprofen sodium hydrate was the most frequently prescribed NSAID, used by 145 of 199 patients and accounting for 72.9% of patients with triple whammy prescriptions (Figure 4B). This was followed by celecoxib (21 patients) and ibuprofen (11 patients). With regard to diuretics, the most commonly prescribed agents were furosemide (41 patients), spironolactone (40 patients), and trichlormethiazide (34 patients) (Figure 4C). These findings regarding NSAIDs and diuretics support the overall prescription trends illustrated in Figure 2. By contrast, a distinct trend was observed for RAS inhibitors, with sacubitril/valsartan sodium hydrate being the most frequently prescribed drug (46 patients), followed by telmisartan (41 patients) and olmesartan medoxomil (30 patients) (Figure 4D).

To summarize the overall prescribing patterns of RAS inhibitors analyzed above, the key proportions are presented in Table 3. Notably, sacubitril/valsartan sodium hydrate accounted for 23% of the RAS inhibitors prescribed to patients with triple whammy prescriptions, significantly higher than its single-agent proportion of 7.4% (*p* < 0.001).

Single-agent and dual-combination prescription counts were analyzed over a 3-year period to minimize annual prescription imbalances. Proportions for triple whammy prescriptions were calculated using the most recent 1-year data to reflect the post-approval year-over-year increase in sacubitril/valsartan prescriptions.

## 4. Discussion

The synergistic pharmacological effects of the triple whammy (consisting of NSAIDs, RAS inhibitors, and diuretics) can increase AKI risk, underscoring the essential role of pharmacists in its prevention. The aims of this study were to investigate prescribing patterns of the triple whammy in Japan using a nationwide patient estimation database and to identify high-risk patient populations.

In previous studies, the focus has primarily been on quantifying the risk of AKI associated with the triple whammy, whereas the aspects of how such prescriptions are formed have not been sufficiently examined. Although real-time monitoring of serum creatinine and urine volume is challenging in routine practice, identifying and avoiding high-risk drug combinations is a more effective strategy for prevention. However, despite these significant epidemiological risks being recognized globally, awareness of the triple whammy among pharmacists remains as low as approximately 30%, indicating insufficient recognition of this issue in clinical practice [19]. By elucidating these nationwide patterns, the present study provides a crucial epidemiological foundation for enhancing patient safety and represents a vital first step toward optimizing medication management. As highlighted by the findings of the present study, prescribers of the triple whammy tend to be biased toward specific drugs, as based on an analysis of recent multi-year data on the number of patients prescribed individual agents, two-drug class combinations, and all possible triple whammy prescriptions. No significant long-term structural changes were observed in the prescribing patterns of each drug class, indicating that the drugs constituting the triple whammy are widely and consistently used in routine clinical practice. Importantly, the overall annual number of triple whammy prescriptions demonstrated a statistically significant downward trend over the study period (*p* = 0.048). This decline may reflect growing awareness among healthcare professionals regarding AKI risks associated with polypharmacy, as well as the implementation of clinical guidelines and safety alerts promoting safer prescribing practices. Conversely, a temporary decline in NSAID prescriptions was specifically observed, which may reflect changes in both the medical care provision system and patterns of healthcare utilization during the COVID-19 pandemic.

Analysis of the single-agent drugs revealed that loxoprofen sodium hydrate accounted for approximately two-thirds of all NSAIDs prescribed, suggesting that the prescribing of this drug class in Japan is dependent on a limited number of agents. However, loxoprofen sodium hydrate is contraindicated in patients with severe renal dysfunction, necessitating cautious clinical judgment when co-prescribing NSAIDs to patients with renal vulnerability. Indeed, loxoprofen has been shown to induce a significantly greater elevation in serum creatinine levels compared to acetaminophen, with an incidence of AKI observed in clinical settings [20]. Research has shown that NSAID use is associated with a 1.38-fold increased risk of AKI, particularly in patients with baseline renal dysfunction [21]. With regard to RAS inhibitors, angiotensin receptor blockers (ARBs) predominated in this study. By contrast, enalapril maleate ranked 12 (1,011,092 patients) and imidapril hydrochloride ranked 17 (316,139 patients) among the angiotensin-converting enzyme (ACE) inhibitors prescribed. This preference for ARBs likely reflects their status as a primary clinical choice owing to their superior tolerability and lower incidence of side effects, such as dry cough. Similarly, fundamental drugs for treating heart failure and hypertension (e.g., furosemide and spironolactone) were the most common diuretics prescribed. The distribution of single-agent drugs appeared to strongly influence the drugs constituting the dual-combination and triple whammy regimens. However, the combination of diuretics and RAS inhibitors was characterized by a high prevalence of sacubitril/valsartan sodium hydrate, which likely reflects the treatment strategies for heart failure. Additionally, sacubitril/valsartan sodium hydrate showed a significantly higher prevalence in triple whammy prescriptions than in single-agent use (23% vs. 7.4%, *p* < 0.001), potentially elevating renal risk when NSAIDs are added in heart failure patients with underlying renal vulnerability. Because real-world triple whammy regimens are highly fragmented, memorizing specific three-drug combinations is impractical. Instead, pharmacists should focus on key driver drugs—such as sacubitril/valsartan or loxoprofen—as clinical triggers for screening and preventing inadvertent triple whammy exposure. Regional prescribing patterns vary considerably, with Swiss data favoring ibuprofen and ACE inhibitors, whereas Japanese practice relies heavily on loxoprofen and ARBs/ARNIs [22]. Although these drug-level differences limit direct international generalizability, reporting detailed local prescribing patterns remains essential to clarify regional safety profiles.

A systematic review and meta-analysis of randomized controlled trials, including Japanese cohorts, reported that sacubitril/valsartan sodium hydrate provides superior long-term renoprotective effects compared to conventional RAS inhibitors [23]. However, it is important to note that the risk of AKI—potentially due to a transient decrease in renal perfusion pressure during the treatment initiation phase—remains comparable to that of conventional RAS inhibitors [24,25]. Furthermore, clinical data have indicated that there were no significant differences in renal outcomes between patients prescribed sacubitril/valsartan and their matched ACE inhibitor or ARB counterparts, regardless of whether they were previously prescribed an ACE inhibitor or ARB [26]. In one study in Japan, sacubitril/valsartan sodium hydrate was discontinued in some patients with heart failure owing to worsening kidney function, suggesting heterogeneity in the renal effects of this drug [27]. Consequently, given the complexity of heart failure, its numerous comorbidities, and the inevitability of polypharmacy, pharmacists must extend their vigilance regarding triple whammy risks to a broader patient population, including those transitioning from other RAS inhibitors. The role of pharmacists in continuous medication management and follow-up is becoming increasingly important to ensure the safety of these complex regimens. As the final component of the triple whammy for patients with heart failure, NSAIDs are likely prescribed in orthopedic or dental departments. In practice, NSAIDs are frequently prescribed across these departments [28,29]. However, the rate of “Separation of Dispensing and Prescribing Functions” is lower in dentistry compared to other medical fields, with many dental clinics continuing to provide in-hospital prescriptions. Consequently, it is not uncommon for pharmacies to be unaware of prescriptions issued by dental practices. Therefore, there is a risk that identifying potential triple whammy combinations may become difficult. In Japan, advancing medical-dental-pharmaceutical collaboration through “tracing reports” is considered a crucial strategy to avoid the triple whammy [30]. A survey based on records from a single pharmacy found that patients with double whammy prescriptions accounted for 3.9% of all patients, indicating that constant vigilance is required in routine clinical practice [31].

This study has several limitations. First, the AHI partners database does not contain clinical laboratory data, such as serum creatinine levels or estimated glomerular filtration rate (eGFR). Consequently, it was not possible to directly evaluate clinical outcomes, such as incidence of AKI following triple whammy. Furthermore, it does not establish a causal relationship between drug combinations and renal dysfunction, as this is a cross-sectional analysis. To validate these findings and establish a causal relationship, future follow-up studies incorporating clinical laboratory data are warranted. Second, medical claims data reflect prescription records and cannot fully capture actual medication adherence or confirm true concomitant use. Therefore, our definition of combined use may have overestimated the prevalence of triple whammy prescribing. Additionally, the database lacks detailed information on specific dosages, dosing periods, and the use of over-the-counter (OTC) NSAIDs, which may have led to an underestimation of the actual exposure. Third, the figures in this study are national estimates derived using correction factors. Therefore, discrepancies between these estimates and clinical practice may occur, particularly in cases of rare diseases or specialized prescription patterns. While actual number of patients were analyzed to avoid extrapolation errors in the triple whammy cohort, the limited sample size for individual medications implies that agent-specific inferences carry inherent uncertainty and should be interpreted as hypothesis-generating. Finally, other potential confounding factors—such as comorbidities and lifestyle habits—were not fully addressed. These limitations should be considered when interpreting the results of this study.

## 5. Conclusions

At the level of individual agents, the diuretics and RAS inhibitors most commonly used were generally standard drugs, whereas loxoprofen was the predominant NSAID. Notably, this study also demonstrated a high prevalence of sacubitril/valsartan sodium hydrate use in combination therapy. In patients with heart failure, diuretics are frequently used in combination with sacubitril/valsartan sodium hydrate-containing regimens, making these patients particularly vulnerable to the “triple whammy.” Although sacubitril/valsartan sodium hydrate is a beneficial treatment for heart failure, it requires careful monitoring. Therefore, inadvertent addition of NSAIDs should be strictly avoided to help reduce potential AKI risks.

## Figures and Tables

**Table 1:** List of single-agent drugs associated with the triple whammy used in the analysis.

Classification	Drugs
NSAIDs	Acemetacin, Ampiroxicam, Amfenac sodium hydrate, Ibuprofen, Indometacin, Indometacin farnesil, Etodolac, Emorfazone, Oxaprozin, Ketoprofen, Zaltoprofen, Diclofenac sodium, Sulindac, Tiaprofenic acid, Tiaramide hydrochloride, Nabumetone, Naproxen, Piroxicam, Flufenamate aluminum, Flurbiprofen, Bucolome, Pranoprofen, Proglumetacin maleate, Mefenamic acid, Meloxicam, Mofezolac, Loxoprofen sodium hydrate, Lobenzarit disodium, Lornoxicam, Celecoxib
RAS inhibitors	Alacepril, Imidapril hydrochloride, Enalapril maleate, Captopril, Quinapril hydrochloride, Cilazapril hydrate, Temocapril hydrochloride, Delapril hydrochloride, Trandolapril, Benazepril hydrochloride, Perindopril erbumine, Lisinopril hydrate, Azilsartan, Irbesartan, Olmesartan medoxomil, Candesartan cilexetil, Telmisartan, Valsartan, Losartan potassium, Sacubitril/valsartan sodium hydrate, Aliskiren fumarate
Diuretics	Esaxerenone, Eplerenone, Spironolactone, Azosemide, Torasemide, Tripamide, Piretanide, Furosemide, Bumetanide, Mefruside, Indapamide, Trichlormethiazide, Hydrochlorothiazide, Benzylhydrochlorothiazide, Tolvaptan, Isosorbide, Meticrane

**Table 2:** List of fixed-dose combination drugs with the triple whammy used in the analysis.

Classification	Drugs
RAS inhibitors + Diuretics	Irbesartan/Trichlormethiazide, Candesartan cilexetil/Hydrochlorothiazide, Telmisartan/Hydrochlorothiazide, Valsartan/Hydrochlorothiazide, Losartan potassium/Hydrochlorothiazide
RAS inhibitors + Ca^2+^ blocker + Diuretics	Telmisartan/Amlodipine besilate/Hydrochlorothiazide

**Table 3:** Proportions of top-ranked RAS inhibitors in single-agent, dual-combination, and triple-whammy prescriptions.

Top	Single-Agent		Dual-Combination				Triple Whammy (Latest Year)	
			RAS Inhibitors/NSAIDs		RAS Inhibitors/Diuretics			
	Drug	%	Drug	%	Drug	%	Drug	%
1	Olmesartan	19.4	Olmesartan	14.3	Sacubitril/valsartan	20.2	Sacubitril/valsartan	23.1
2	Telmisartan	15.9	Candesartan	13.6	Olmesartan	10.6	Telmisartan	20.6
3	Candesartan	15.2	Azilsartan	11.4	Enalapril	10.4	Olmesartan	15.1
4	Azilsartan	15.0	Telmisartan	10.6	Azilsartan	9.14	Azilsartan	11.6
5	Valsartan	8.81	Valsartan	8.59	Candesartan	8.57	Candesartan	7.04
6	Sacubitril/valsartan	7.41	Irbesartan/ Amlodipine	4.97	Telmisartan	7.38	Enalapril	6.53
7	Losartan	5.95	Telmisartan/ Amlodipine	4.79	Valsartan	5.32	Valsartan	4.02
8	Enalapril	4.70	Candesartan/ Amlodipine	4.63	Irbesartan/ Amlodipine	4.31	Losartan	4.02
9	Irbesartan	4.55	Azilsartan/ Amlodipine	4.32	Azilsartan/ Amlodipine	3.81	Irbesartan	0.50
10	Imidapril	1.47	Sacubitril/valsartan	4.22	Telmisartan/ Amlodipine	3.56	Imidapril	0.50

## Data Availability

The datasets presented in this article are not readily available because the data are proprietary and used under a license from AHI Partners Inc., and are therefore restricted owing to commercial confidentiality and legal limitations. Requests to access the datasets should be directed to corresponding author.

## Abbreviations

The following abbreviations are used in this manuscript:

AKI	Acute Kidney Injury
Scr	Serum creatinine
NSAIDs	Non-Steroidal Anti-Inflammatory Drugs
RAS	Renin–Angiotensin System
MHLW	Ministry of Health, Labour and Welfare
ARB	Angiotensin Receptor Blockers
ACE	Angiotensin-Converting Enzyme
ATC	Anatomical Therapeutic Chemical
CIs	Confidence Intervals
COVID-19	Coronavirus disease 2019
eGFR	estimated Glomerular Filtration Rate
OTC	Over-The-Counter
